# *Green*-Synthesized Zinc Oxide–Bacterial Cellulose Composites: Eco-Friendly Antibacterial Wound Dressings for Faster Healing

**DOI:** 10.3390/polym18091050

**Published:** 2026-04-26

**Authors:** Iuliana-Mihaela Deleanu, Sorana-Gabriela Ivanescu, Gabriela-Olimpia Isopencu, Ovidiu-Cristian Oprea, Mihaela Bacalum, Diana-Lavinia Stan, Sorin-Ion Jinga, Cristina Busuioc

**Affiliations:** 1 Faculty of Chemical Engineering and Biotechnology, National University of Science and Technology Politehnica Bucharest, RO-060042 Bucharest, Romania; iuliana.deleanu@upb.ro (I.-M.D.); ovidiu.oprea@upb.ro (O.-C.O.); 2 Faculty of Medical Engineering, National University of Science and Technology Politehnica Bucharest, RO-060042 Bucharest, Romaniasorin.jinga@upb.com (S.-I.J.); 3 National Center of Micro and Nanomaterials, National University of Science and Technology Politehnica Bucharest, RO-060042 Bucharest, Romania; 4 Horia Hulubei National Institute of Physics and Nuclear Engineering, RO-077125 Magurele, Romania; mihaela.bacalum@nipne.ro (M.B.);

**Keywords:** bacterial cellulose, zinc oxide, plant extract, *green* synthesis, antibacterial properties, wound dressings

## Abstract

The present work aimed to obtain antibacterial wound dressings using bacterial cellulose (BC) as a support, to improve wound treatment and reduce the incidence of infections. To enhance the antibacterial activity of the synthesized dressings, the introduction of ZnO nanoparticles into the BC network by precipitation was pursued. The method chosen to develop ZnO NPs was *green* synthesis, an ecological and sustainable method for obtaining nanomaterials using plant extracts as reducing agents or stabilizers. Thus, the chosen plants were *Ginger rhizomes*, *Bay leaves*, and *Rose hips*, in both fresh and dry form, due to the natural benefits they possess, and the Soxhlet method was used to obtain the plant extracts desired to be used in the synthesis. The composite dressings were developed in two distinct sample series, differentiated by the immersion time of BC in the precursor Zn^2+^ solution. The samples in the first series were obtained by precipitation in a mixture of Zn^2+^ solution and natural extract, whereas the samples in the second series were obtained by successive immersion in Zn^2+^ solution and then in natural extract, which demonstrated a considerable difference. The best antimicrobial activity tested against *Gram*-negative bacterium *Escherichia coli* was recorded for the composite material obtained in the presence of fresh rose hip extract, an aspect most likely related to the morphological and crystalline features of the ZnO phase, but also to the phytochemical profile of the extract used. Such eco-friendly materials represent valuable candidates for wound dressing applications due to their ability to support wound healing, relief burns, and skin irritation, provide antimicrobial protection, promote skin regeneration and reduce scarring, protect sensitive skin, and act as a barrier against external contaminants.

## 1. Introduction

With the need to develop new effective therapeutic options, metal and metal oxide nanoparticles (NPs) have emerged as sustainable alternatives for drug delivery, wound healing, or tissue engineering [1]. Through assessing the numerous NPs investigated, zinc oxide (ZnO) NPs in particular have been proved to be among the most suitable for biomedical and pharmaceutical applications, possessing distinctive physicochemical properties: antimicrobial efficacy against bacteria, viruses, and fungi, anti-inflammatory and antioxidant activities, photochemical stability, minimal cytotoxicity, and cost-effectiveness [2,3]. Besides being already generally recognized as safe, “GRAS”, by the Food and Drug Administration (FDA) [4], unlike other metal oxides, ZnO presents biodegradability (into Zn^2+^ ions) by releasing reactive oxygen species (ROS) on its surface [5,6].

Zinc (Zn), as a chemical element, is naturally essential in skin health and skin healing processes. Therefore, ZnO NPs, having the attributes enumerated before, could sustain angiogenesis and cell proliferation on one hand and could actively reduce the risk of infections on the other hand [7]. ZnO NPs could be produced by various methods, like sol–gel synthesis, vapour-phase synthesis, hydrothermal or mechanochemical routes, and more. It is, however, widely accepted that specific functionalities of ZnO NPs, related to size, morphology, and/or crystallinity, depend on the synthesis parameters and post synthesis approaches [8].

Simpler and less toxic alternatives to produce biocompatible ZnO NPs are investigated, and among them, the biological routes are considered as the most promising for the future. Designated as *green* routes, the techniques involving plants or plant derivatives, microorganisms (fungi, yeasts or bacteria), or algae allow for the production of *biogenic* ZnO. Plant-mediated synthesis, for instance, allows the successful preparation of highly functional nanostructures as the result of antibacterial capabilities of plant extract cumulated with NPs’ properties [9]. Furthermore, the bioactive components of plant extracts promote bioreduction, acting like reduction and stabilizing agents in NP formation [10]. The only drawbacks, so far, of plant-mediated NP synthesis remain the scalability and reproducibility because of inherent batch-to-batch variability and difficulties in optimization of the synthesis parameters [11].

This work investigates, qualitatively, the in situ development of biogenic ZnO NPs composited with bacterial cellulose to be used as wound dressings for faster healing. Bacterial cellulose (BC), a nearly pure cellulose produced by microbial routes in the form of three-dimensional porous fibrillar nanostructures, was employed as the support material. Its unique inherent properties made BC a most promising biopolymer in wound healing and tissue regeneration applications [12,13]. Three different kinds of vegetal materials were used to prepare the extracts to be used as synthesis mediums: *Ginger rhizomes*, *Bay leaves*, and *Rose hip pericarp*.

*Zingiber officinale*, known as ginger, often used as a food condiment and valued in traditional medicine, is also widely used in *green* ZnO NPs synthesis. Aqueous rhizome extracts proved efficient due to natural properties and constituents (including gingerols, shogaols, and volatile oils), mediating NP biosynthesis and offering particles stability [14,15,16,17,18,19].

*Laurus nobilis* (bay), another medicinal and aromatic plant used around the world, can mediate the fabrication of ZnO NPs, as previously reported. Bay leaf extracts were successfully used for this purpose, as contain numerous bioactive compounds (essential oils, sesquiterpene lactones, flavonoids, and alkaloids) assist in the reducing, stabilization, and capping processes [20,21,22,23,24].

In contrast, the usage of rose hip (*Rosa canina*) in the biofabrication of ZnO NPs was barely investigated. However, its potential was demonstrated, owing to the high content of vitamin C, flavonoids, and phenolic compounds. Fruit extract (in whole) and seeds extract assisted ZnO NPs’ biofabrication as an eco-friendly approach to provide antimicrobial ability and low toxicity [25,26].

The novelty of this study lies in the development of a bio-based composite material by in situ deposition of ZnO on BC through a *green* synthesis approach, using a variety of plant extracts derived from *Ginger rhizomes*, *Bay leaves*, and *Rose hips*, in both fresh and dried forms, with extraction performed via the Soxhlet method. To impart antibacterial properties, ZnO NPs were incorporated into the membrane, together with natural bioactive compounds of the selected plants. This approach allows the modulation of nanoparticle formation through a diverse phytochemical profile, which leads to improved control over particle size, morphology and functional properties. Furthermore, the incorporation of ZnO into a BC matrix improves the applicability of the material, providing a sustainable biodegradable support with high surface area and mechanical stability. The resulting composite is expected to exhibit improved biofunctional performances compared to systems based on individual extracts or conventional polymer matrices, highlighting its potential for the development of biocompatible wound dressings for skin injuries.

## 2. Materials and Methods

### 2.1. Materials

For the manufacturing of the wound dressings with improved properties, the following materials and reagents were employed: BC membranes, plant (*Ginger rhizomes*, *Bay leaves*, and *Rose hips*) extracts, both obtained in our laboratories, zinc acetate dihydrate (Zn(OAc)_2_·2H_2_O, *M_w_* = 219.5 g/mol, ≥98%, Sigma-Aldrich, St. Louis, MO, USA), and ammonium hydroxide (NH_4_OH, 25%, Sigma-Aldrich, St. Louis, MO, USA).

### 2.2. Synthesis Methods

#### 2.2.1. Production and Purification of Bacterial Cellulose Membranes

BC was obtained in the Microbiology Laboratory of Chemical and Biochemical Engineering, Department of National University of Science and Technology Politehnica, Bucharest, following procedures previously described elsewhere [27,28]. Briefly, BC was synthetized in static culture conditions and harvested after 7 days as white gel-like pellicles, formed at the air–liquid interface. The cultivation was performed on modified Hestrin–Schramm medium (containing 20 g/L fructose as carbon source) in controlled conditions (27 °C, atmospheric pressure). The medium was sterilized (autoclaved at 121 °C for 20 min) and then inoculated. *Komagataeibacter xylinus*, isolated from apple cider vinegar [29], was used as inoculum (at a 1:10 *v*/*v* ratio), being known as a highly productive bacteria [30]. The pellicles were purified by boiling them in 0.1 M NaOH aqueous solution for 60 min and then washed several times with deionized water until neutral pH. Purified BC sheets, approximately 10 mm thick (determined with a Dial Thickness Gauge Gage Measuring Tool 0–20 mm, Hoffmann Group, Munich, Germany), were further used as wet membranes.

#### 2.2.2. Plant Extracts Preparation

Three different vegetal materials were purchased from a local market in Bucharest and used in this study: ginger (*Zingiber officinale* Roscoe) rhizomes, bay (*Laurus nobilis* L.) with fully developed leaves, and ripened *Rose hips* (fruits of *Rosa canina* L.). The fresh plant parts were cleaned with distilled water and minimally processed: the *Ginger rhizomes* were peeled, the midrib and petiole of the leaves were removed, and so were the seeds of the *Rose hips*. Depending on the purpose, wet fresh materials were directly refrigerated until their processing (at 4 ± 1 °C) or dried at 50 °C in a food dehydrator (Tribest Sedona Express SDE-P6280, Anaheim, CA, USA) to constant weight prior to refrigeration. The moisture content of fresh and dried samples was determined at 120 °C (Ohaus MB32 Moisture Analyzer, Fisher Scientific, Hampton, NH, USA).

**Figure 1:**
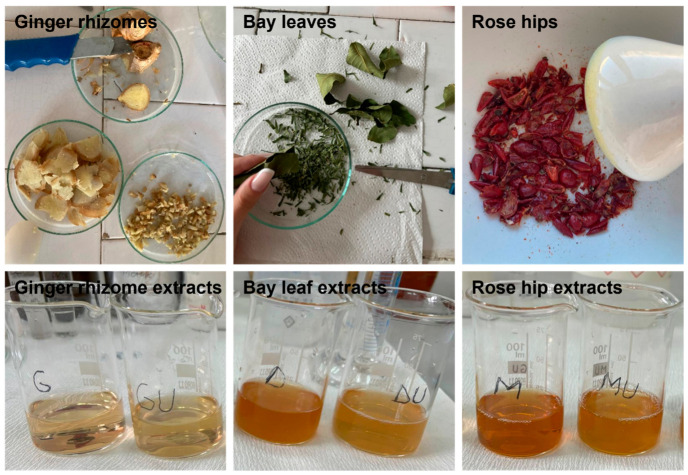
Representative digital images of the plants (*Ginger rhizomes*, *Bay leaves*, and *Rose hips*) used in this study (**upper row**), and of the corresponding extracts obtained via the Soxhlet method (**lower row**).

To prepare the plant extracts, the conventional Soxhlet equipment was used to enable improved extraction efficiency [31]. All samples were ground using a domestic coffee mill to obtain fine solid particles. The coarse particles were removed using a vibratory sieve shaker (AS 200 basic, Retsch GmbH, Haan, Germany), while particles having diameters lower than 0.2 mm were used. The main material and operational parameters applied in the extraction experiments are presented in Table 1. All experiments were conducted with water as the extraction solvent for a total 4 h of extraction time. In the end, six types of extracts were obtained: *Ginger rhizomes* (fresh and dried), *Bay leaves* (fresh and dried), and *Rose hips* (fresh and dried). Representative images of the vegetal specimens and final extracts are shown in Figure 1.

#### 2.2.3. Powders Synthesis

To obtain 500 mL of a 0.5 M Zn^2+^ solution, 54.875 g of Zn(OAc)_2_·2H_2_O was weighed and used as a precursor, being dissolved in an appropriate volume of distilled water. For the synthesis of ZnO powders, the previously described plant extracts were used as precipitating and capping agents under similar conditions. In all cases, 10 mL of Zn(OAc)_2_·2H_2_O aqueous solution was mixed with 10 mL of plant extract (1:1 *v*/*v* ratio). All mixtures were heated on a hot plate at 100 °C under constant stirring. The initial pH was approximately 6 and was adjusted using a 25% ammonia solution (several drops). Precipitation occurred at pH 7.

**Figure 2:**
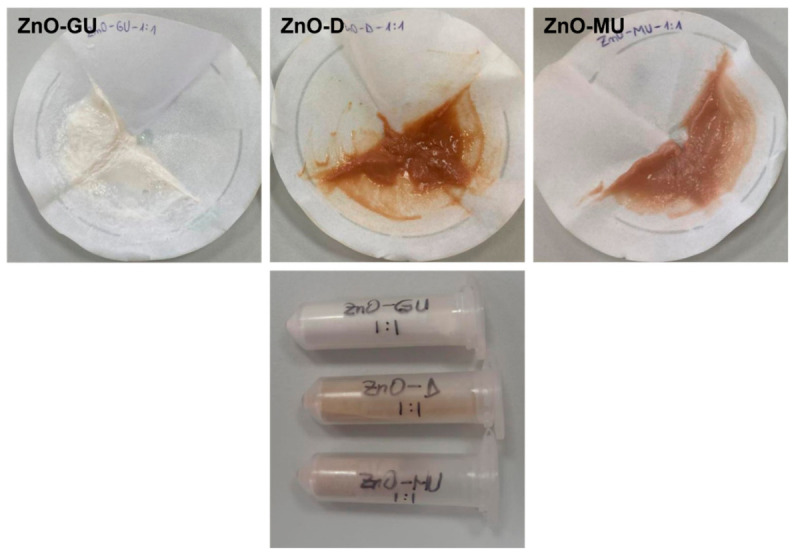
Representative digital images of three obtained precipitates (**upper row**) and the corresponding final powders (**lower row**), illustrating differences in coloration associated with the use of different plant extracts.

At the moment of precipitation, each solution, initially ranging in colour from light brown to dark brown, became significantly more whitish compared to its initial state and acquired a gelatinous texture. The next step involved filtering the obtained precipitate and washing it with distilled water, as displayed in Figure 2. Finally, the sample was dried in an oven at 100 °C for 48 h. Representative powders obtained after this stage are presented in Figure 2. These were named according to the extract used for synthesis: ***ZnO-G*** and ***ZnO-GU*** for fresh and dried *Ginger rhizomes*, ***ZnO-D*** and ***ZnO-DU*** for fresh and dried *Bay leaves*, and ***ZnO-M*** and ***ZnO-MU*** for fresh and dried *Rose hips*, respectively.

#### 2.2.4. Obtaining Composite Membranes by Single Immersion

The first series of composite samples was prepared by impregnating the BC membrane with ZnO NPs and compounds from the plant extracts, with BC serving as a support matrix for the incorporation of the antibacterial phases. BC was cut into cuboids measuring 2 × 2 × 1 cm, and three such samples were used for each experiment.

The impregnation method involved immersion. BC was placed in a beaker with 30 mL of Zn^2+^ solution and heated on a hot plate at 100 °C under magnetic stirring for 30 min. After this, 30 mL of extract was added, and the mixture was allowed to homogenize for 30 min. Next, the pH was adjusted by adding 15 drops of 25% NH_4_OH solution, followed by an additional 30 min stirring period. Finally, the modified BC specimens were removed from the solution and placed on parchment paper in Petri dishes. The last step involved drying the impregnated BC samples in an oven at 100 °C for 48 h, replacing the parchment paper whenever it became excessively wet. This procedure was carried out for each type of extract, resulting in a total of six different samples, with the following notations: ***BC-ZnO-G*** and ***BC-ZnO-GU*** for fresh and dried *Ginger rhizomes*, ***BC-ZnO-D*** and ***BC-ZnO-DU*** for fresh and dried *Bay leaves*, and ***BC-ZnO-M*** and ***BC-ZnO-MU*** for fresh and dried *Rose hips*, respectively. Pure BC (***BC***) was also included as a control during the drying stage.

#### 2.2.5. Obtaining Composite Membranes by Successive Immersion

The second series of composite samples was also prepared by immersion, but with a modification in the duration that BC remained in the Zn^2+^ solution. Thus, BC specimens were immersed in 30 mL of Zn^2+^ solution and left under magnetic stirring for 24 h, aiming to maximize the attachment of Zn^2+^ ions to the surface of the BC membrane. After 24 h, BC swelled, and the Zn^2+^ solution turned whitish due to the release of some fibrils.

In the next step, 30 mL of extract were placed in a beaker, and 15 drops of 25% NH_4_OH solution were added to adjust the pH. The beaker was then placed on a hot plate at 100 °C under magnetic stirring for 30 min, during which the solution colour changed: the rose hip extract turned crimson, the bay leaf extract changed to reddish-brown, while the ginger extract showed no significant colour change.

**Figure 3:**
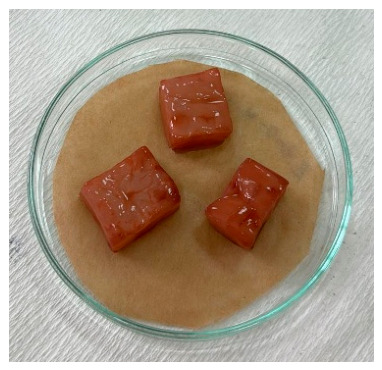
Representative digital images of BC-ZnO-M specimens before the drying step, showing noticeable colour variations compared to the white appearance of pure BC.

The BC specimens were removed from the Zn^2+^ solution and placed into the beakers with extract and NH_4_OH, then stirred for 60 min. No significant changes were observed during this period. The final step was the same as in the case of the first series: the impregnated BC samples were placed on parchment paper and dried in an oven at 100 °C for 48 h. It was observed that BC immersed in bay leaf and rose hip extracts acquired a reddish colour, with a more intense hue for the extracts made from fresh plants compared to dried ones, as displayed in Figure 3.

### 2.3. Characterization Methods

#### 2.3.1. Physicochemical Characterization

Morphological investigation through scanning electron microscopy (SEM) and compositional analysis by energy dispersive X-ray (EDX) spectroscopy were performed with a FEI Quanta Inspect F50 microscope (FEI Company, Hillsboro, OR, USA) operated at an accelerating voltage of 30 kV, spot size 3.5, and a working distance of 10 mm. The samples were previously coated with a layer of gold by direct current magnetron sputtering for 60 s.

The bonds and groups present in the samples were assessed with the help of Fourier-transform infrared (FTIR) spectroscopy, by using a Thermo Scientific Nicolet iS50 spectrophotometer (Thermo Fisher Scientific, Waltham, MA, USA) with a wavenumber range of 400–4000 cm^−1^, a resolution of 4 cm^−1^, and 32 scans per sample.

X-ray diffraction (XRD) allows for qualitative and quantitative analysis of crystalline phases, thus determining the degree of crystallinity and crystallite size. The evaluation was performed using a Shimadzu XRD 6000 diffractometer (Shimadzu Corporation, Kyoto, Japan) with Ni-filtered Cu K*α* radiation (*λ* = 0.154 nm), 2*θ* ranging between 20 and 80°, scanning speed of 2°/min, step of 0.02°, and preset time of 0.6. The most common method for calculating crystallite size from the XRD results is the Scherrer equation:

$$D=\frac{K\times \lambda }{\beta \times cos\theta }$$

where *D* is the average crystallite size (nm), *K* is a shape factor (usually 0.9 for spherical particles), *λ* is the X-ray wavelength (0.15406 nm for Cu K*α*), *β* is the full width at half maximum (*FWHM*, in rad) and *θ* is the Bragg angle (°).

Thermogravimetric analysis (TGA) was performed with a NETZSCH STA 449 F3 Jupiter equipment (NETZSCH GmbH, Selb, Germany), from room temperature to 900 °C, with a heating rate of 10 °C/min, in air, to determine the composition of the samples and their thermostability.

To determine the swelling degree, the samples were dried to constant weight and immersed in distilled water at room temperature (20 °C). The experiments were conducted in triplicates, and the measurements were performed until constant mass of swollen samples (24 h). Swelling degree (*SD*, %) was obtained by measuring the initial weight (*m_i_*, g) and the weight in swollen state (*m_s_*, g), using the following equation:

$$SD=\frac{\left(m_{s}-m_{i}\right)}{m_{i}}\times 100$$

#### 2.3.2. Antibacterial Assay

Regarding the antimicrobial activity, *Gram*-negative bacterium *Escherichia coli* (DH5K strain) from the microorganism collection of the Bioreactor Laboratory was used in this study. The culture medium was Luria–Bertani (modified Miller) agar (purchased from Roth, Karlsruhe, Germany, 2024), a nutrient-rich medium widely used for standard *E. coli* cultivation. Plates were inoculated with 100 µL of bacterial suspension (McFarland 0.5) using the inoculum depletion method and left for ~1 h in a humidity-controlled oven to allow uniform absorption of the suspension and to prevent excess liquid. The samples were cut into 6 mm discs, with triplicates prepared for each sample, and then sterilized under UV light (256 nm) using a portable UV lamp (ROTH type IV, 254/366 nm) for 30 min, weighed if necessary, and aseptically placed on the surface of the medium. The plates were then incubated at 37 °C for 24 h. Antimicrobial activity was evaluated by measuring the zone of inhibition (*IZ*, mm), corresponding to the clear area around the disc where bacterial growth was completely inhibited, and the hallow zone (*IH*, mm), indicating areas where bacterial growth was reduced but not fully stopped. These indicators were adjusted according to the sample weight.

#### 2.3.3. Cell Morphology

L929 fibroblast cells (ATCC, Manassas, VA, USA) were used to evaluate the samples’ biocompatibility. Cells were cultured in Dulbecco’s Modified Eagle’s Medium (DMEM), supplemented with 10% fetal bovine serum (FBS) and 1% penicillin/streptomycin (Thermo Fisher Scientific, Waltham, MA, USA), in a humidified incubator at 37 °C and 5% CO_2_. Cell morphology was evaluated by fluorescence microscopy. The cell nucleus and cytoskeleton were fixed using a 3% paraformaldehyde solution, permeabilized with 0.1% Triton X-100. Then, the actin filaments were stained with FITC-phalloidin (Thermo Fisher Scientific, Waltham, MA, USA) and nuclei with Hoechst 33342 (Thermo Fisher Scientific, Waltham, MA, USA). Finally, slides were mounted using FluorSaveTM and imaged using an Olympus BX-51 epifluorescence microscope equipped with a 40× objective and DAPI/Hoechst and GFP/FITC filters.

#### 2.3.4. Statistical Analysis

Microsoft^®^ Excel^®^ for Microsoft 365 MSO and one-way analysis of variance (ANOVA, *p* < 0.05) were used to perform the statistical analysis. All experiments were performed in triplicates, unless otherwise stated, and all the results are presented as *mean ± standard deviation*.

## 3. Results and Discussion

### 3.1. Powders Characterization

**Figure 4:**
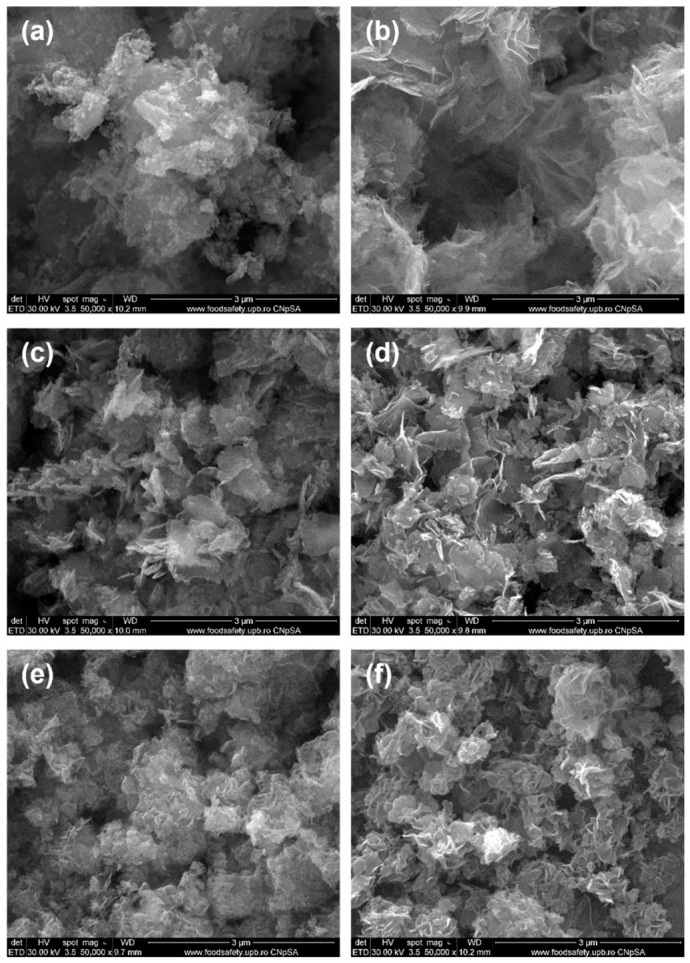
SEM images at 50,000× magnification of *green*-synthesized ZnO powders: (**a**) ZnO-G, (**b**) ZnO-GU, (**c**) ZnO-D, (**d**) ZnO-DU, (**e**) ZnO-M, and (**f**) ZnO-MU.

In Figure 4, SEM images of ZnO powders synthesized in the presence of fresh and dry plant extracts by precipitation are shown. In general, the particles appear fairly heterogeneous and agglomerated, predominantly exhibiting flake-like or folded morphologies with relatively well-defined edges, resembling petals. Their thickness lies within the nanometric range, although their surface area is difficult to estimate. Fluffy micrometric aggregates are formed from these two-dimensional entities, giving the impression of a dense morphology. No significant differences were observed between the morphologies of powders obtained using fresh or dry plants. However, ginger appears to promote a less controlled synthesis: either fine quasi-spherical particles (~20 nm) or large spheres composed of tightly packed nanofoils can be achieved, depending on whether the plant used is fresh or dry (Figure 4a,b). In contrast, *Rose hips* lead to the most uniform nanoscale morphology, with an exfoliated appearance, suggesting strong interactions between Zn^2+^ cations and the compounds present in the extract. Such interactions promote enhanced nucleation while limiting particle growth. The corresponding SEM images reveal either tightly folded nanosheets or loosely stacked flake-like aggregates, with a slightly more textured morphology in the latter case (Figure 4e,f)). Among all the plant extracts studied, the one produced from *Rose hips* proves to be the most effective capping agent, preventing uncontrolled growth and aggregation, and stabilizing particle size and shape. In the case of ZnO NPs synthesized using bay leaf extract, in addition to the hexagonal shape, the flower petal appearance, as reported in the literature [32], is also present here (Figure 4c,d).

In the article by Aliannezhadi et al. [33], it was demonstrated that the crystallite size of ZnO NPs synthesized via a *green* method increases with increasing ginger extract concentration. A low concentration of ginger extract leads to the formation of ZnO nanoflakes, whereas a flower-like structure gradually develops as the extract concentration increases. The ZnO-G sample exhibits a morphology different from that reported in the reference article, whereas the ZnO-GU sample closely resembles it, displaying the same flake-like, fluffy morphology.

In the article by Jun Xu et al. [34], the formation of ZnO NPs with plant extracts such as *Aloe vera*, *Allium sativum* (garlic), *Rosmarinus officinalis* (rosemary), *Ocimum basilicum* (basil), *Camellia sinensis* (green tea), and others was investigated. The resulting ZnO NPs exhibited diverse morphologies, including flower-like, triangular, hexagonal, and spherical shapes. The article also discusses various factors that can influence the synthesis of ZnO NPs, such as extract concentration, with higher extract concentration leading to smaller particle sizes, precursor concentration, where high concentration can change particle shape from spherical to cylindrical, reaction time, and the pH of the solution. The samples obtained in this study contain ZnO NPS with morphologies comparable to those reported in the reference article, exhibiting various shapes and sizes depending on the interaction with the plant extract used. Among these, flower-like and flake-like structures are the most frequently formed during the ZnO nanoparticle synthesis process.

Given the above-mentioned points, it can be concluded that NP formation reproducibility needs precise control of numerous operational and technical parameters: composition and concentrations of extracts and metal precursors, materials’ amounts and volumes, pH, temperature, mixing and reaction time. Any slight change could determine significant differences in NPs characteristics [35]. Therefore, the findings of numerous reported studies are valid in certain conditions and only at the laboratory level. As a direct consequence, the scalability remains challenging, depending not only on the nature of the plant, but also on cultivation, processing, and synthesis protocols. As such, both reproducibility and scalability of NPs are hindered by several factors which are difficult to address. Firstly, plant raw material composition is strongly influenced by genetics, soil quality, and variable climate conditions; additionally, the plant quality, purity, and stability must be guaranteed through regulated and well documented agricultural practices (from cultivation to storage) [36,37]. Secondly, different processing techniques for biological agents and plant extracts’ preparation need strict standardization protocols to provide consistent physicochemical properties, especially at large production scales when operational issues often occur (difficulties in maintaining temperature, pH, mixing) [38,39].

In another study [32], ZnO NPs were synthesized, both by chemical methods and via *green* synthesis using plant extracts, specifically *Bay leaves* and cinnamon. It is noteworthy that NPs obtained through *green* synthesis exhibit minimal aggregation, likely due to the stabilizing effect of compounds in the plant extract acting as natural reducing, caping, and stabilizing agents according to the recent literature [40,41].

Although hard to predict or scale, specific properties like morphology, surface characteristics, crystallinity, or biological activities of biogenic ZnO NPs depend on the plant extract nature and composition. Said et al. [42] suggested that NP nucleation and growth kinetics could be influenced by the surface-bound organic functional groups (like polyphenols, flavonoids, and saponins) present in the extracts. However, the extracts are highly complex systems, containing various secondary metabolites, and the synthesis mechanisms remain incompletely understood [43,44]. In general, the synthesis mechanism is considered to have three main stages: (i) the reduction and stabilization stage, when Zn^2+^ ions and the aromatic hydroxyl groups of biomolecules are forming a stable complex; (ii) hydrolysis stage, when the complex transforms into Zn(OH)_2_; (iii) thermal treatment to convert the hydroxide into ZnO and remove phytochemicals. In some cases, as in the case of this study, biomolecules’ presence can be detected on the surface of the ZnO NPs even after calcination, proving caping effect and preventing agglomeration [44,45,46].

**Figure 5:**
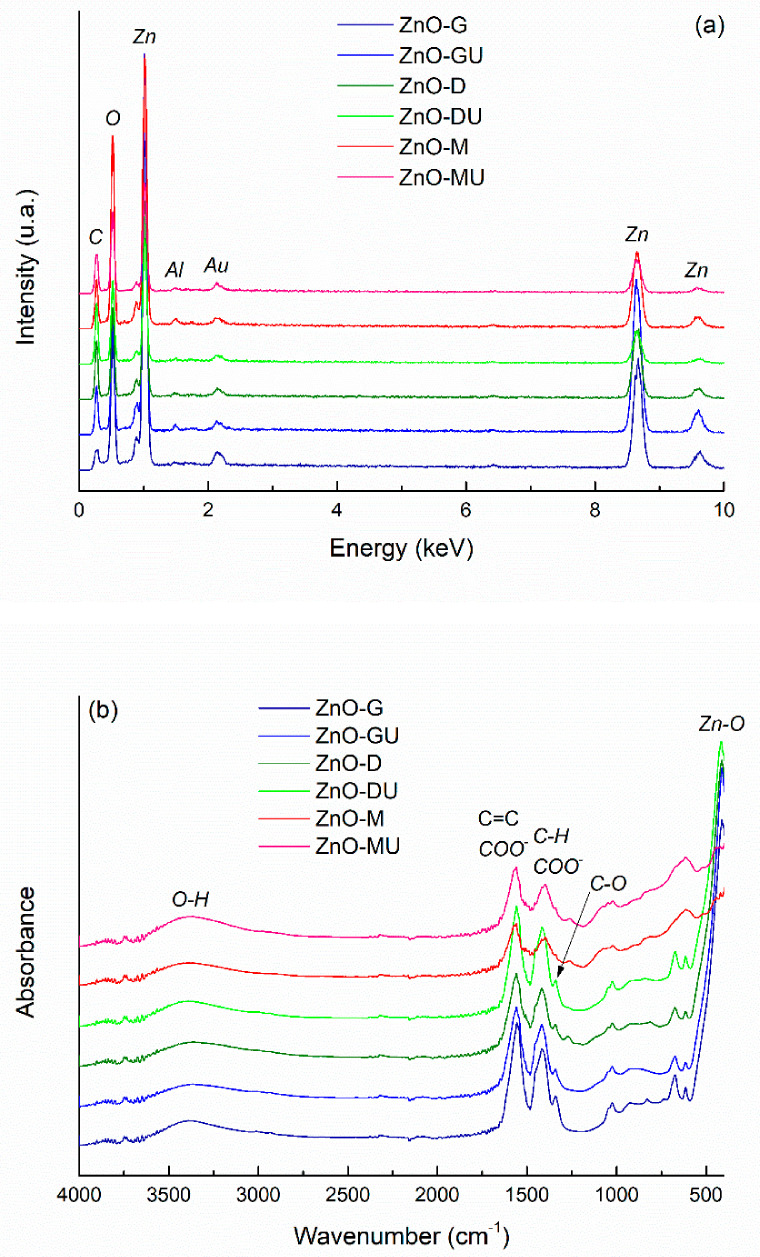
(**a**) EDX spectra and (**b**) FTIR spectra of *green*-synthesized ZnO powders, highlighting the presence of Zn and O elements and the characteristic vibrational bands associated with ZnO and residual organic compounds from the extracts.

The EDX spectra of the *green*-synthesized ZnO powders are presented in Figure 5a, showing the presence of characteristic elemental peaks and indicating that the intensity is similar across the different samples. It is noteworthy that, although different plant extracts were used, all samples contain the same main elements, namely zinc (Zn) and oxygen (O). The presence of carbon (C) is attributed to residues from the plant extracts, as its intensity is too high to originate solely from the carbon tape used for sample mounting. Gold (Au) is observed due to the coating applied to the samples, while the weak signal for aluminum (Al) comes from the stub on which the samples were mounted.

Figure 5b shows the FTIR spectra of ZnO powders with residual plant extracts used in the *green* synthesis. The samples exhibit similar absorption peaks, indicating the presence of the same chemical bonds in powders synthesized with different plant extracts. The most intense vibrational band in the 400–700 cm^−1^ range corresponds to Zn–O bonds. The weak maxima located between 1030 cm^−1^ and 1340 cm^−1^ can have several explanations: C–O stretching vibrations from phenolic, alcoholic, or ether groups; C–N stretching or O–H bending vibrations of phenolic compounds in the plant extract, all confirming the presence of biomolecules on ZnO surface, that act as natural capping agents. The vibrational band with maximum at ~1400 cm^−1^ is associated with symmetric carboxylate (COO^−^) groups stretching or C–H bonds bending in alkyl groups, while the signal located at ~1565 cm^−1^ corresponds to the stretching vibrations of aromatic C=C bonds or carboxylate (COO^−^) groups from plant compounds. The broad band centred at ~3400 cm^−1^ is attributed to the stretching vibrations of hydroxyl (OH^−^) groups, indicating the presence of hydroxyl groups from plant biomolecules or adsorbed water on ZnO surface. The only spectra that stand out are those of ZnO powders obtained in the presence of rose hip extracts (fresh and dry), for which the dominant band is significantly attenuated, both in intensity and sharpness. This may suggest a different crystallization behaviour, probably a less ordered crystal structure, resulting in a lower crystallinity or a partially amorphous nature compared to ZnO obtained with other plant extracts [47,48]. Overall, the spectra indicate that plant-derived compounds not only cap and stabilize the NPs but also preserve characteristic functional groups on their surface [49].

**Figure 6:**
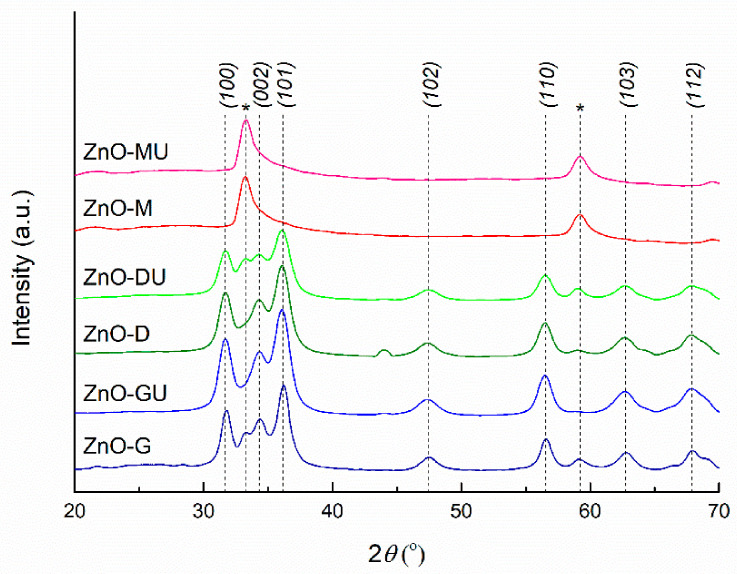
XRD patterns of *green*-synthesized ZnO powders, indicating their crystalline nature and the presence of characteristic reflections corresponding to the wurtzite phase. * represents additional diffraction peaks that are not typical of ZnO.

The XRD patterns of ZnO powders synthesized with plant extracts are presented in Figure 6, showing the characteristic diffraction peaks of ZnO. The three main peaks are observed at ~31°, ~34°, and ~36°, corresponding to the Miller indices (100), (002), and (101), followed by peaks indexed as (102), (110), (103), and (112) at ~47°, ~56°, ~62°, and ~67°, respectively. Moreover, the peak width indicates a low degree of crystallinity. Thus, the prepared ZnO exhibits a hexagonal wurtzite crystal structure, the P63mc space group, in agreement with ICDD 00-080-0075 [50].

From the graphical representation, it is evident that powders synthesized with ginger rhizome and bay leaf extracts exhibit the most well-defined diffraction peaks associated with ZnO, whereas powders prepared with rose hip extract show a different diffraction pattern compared to the others, with diffraction peaks at ~33° and ~59°. Several explanations can be proposed: the presence of peculiar biomolecules in the rose hip extract influences nucleation and crystal growth, possibly leading to partially amorphous or highly nanostructured ZnO particles; the emergence of nanocrystals with preferential orientation; the presence of a zinc oxihydroxide phase [51], rather than pure ZnO; or the formation of a non-stoichiometric oxide with oxygen vacancies in the crystal lattice. These observations are in good agreement with the previous FTIR analysis, which also highlighted distinct bonding characteristics for the two powders prepared with rose hip extracts.

The average crystallite size was determined for the initial samples presented in Figure 6, whereas the last two were excluded due to their atypical diffraction patterns, which deviate from that of well-crystallized ZnO. All calculated values fall within the 6–8 nm range, the largest crystallite size being associated with the sample synthesized using fresh ginger rhizome extract. The values are very close to each other, and the observed differences are not statistically significant, indicating minimal variation between samples. These absolute values also suggest a relatively low degree of crystallinity, consistent with the formation of small nanocrystalline domains of ZnO.

### 3.2. Bacterial Cellulose–Zinc Oxide Composites Characterization

In the medical field, among the first applications of BC membranes was their use as temporary skin replacement in burned wounds [52]. Although BC is “generally recognized as safe (GRAS)” by FDA since 1992, its applicability on a commercial scale remained limited till this day. The existing market addressing BC-based wound dressing and healing is limited to, more or less, 10–15 products. Nevertheless, these products offer limited bioactivity, with most of them being used to control exudates, protect wounds, and block pathogens [53,54]. BC membranes alone allow perspiration and gas (O_2_/CO_2_) exchange, reduce electrolytes and protein loss, and favour faster healing in comparison with traditional materials like gauze [55]. Membracel^®^ (Vuelo Pharma, Curitiba, Brazil) for example, a pure BC membrane industrially produced for this purpose, can be considered as a reference in comparative studies. In a comprehensive study, Palacio et al. [56] characterized Membracel^®^ and evaluated it. Thus, analyzed using SEM, Membracel^®^ presented regular, smooth surface and low fibre density.

**Figure 7:**
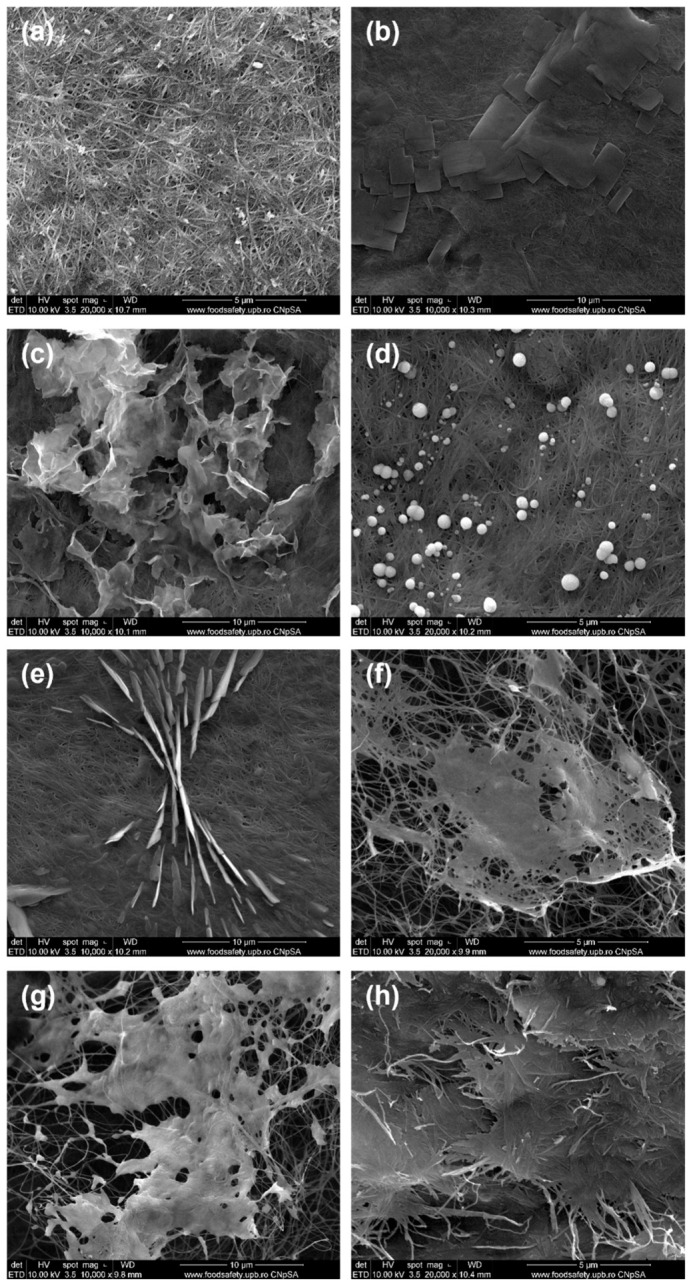
SEM images at 10,000× or 20,000× magnification of the samples in the first series: (**a**) BC, (**b**) BC-ZnO-GU, (**c**,**d**) BC-ZnO-G, (**e**) BC-ZnO-D, (**f**) BC-ZnO-DU, (**g**) BC-ZnO-M, and (**h**) BC-ZnO-MU.

The aspects of the BC membrane, used in this study as a support for the intended antibacterial dressings, is shown in Figure 7a through SEM images. This reveals a dense and porous, and, at the same time, three-dimensional network of interconnected fibres. These are organized like a mesh and randomly distributed, with nanometric dimensions ranging from 20 nm to 100 nm. The denser structure can be related to the preparation and purification procedures, carefully applied and controlled at the laboratory scale, compared to industrial production. The images from Figure 7b–h are representative for the first series of samples, those obtained by single immersion in a solution containing the zinc precursor and different plant extracts. In the case of fresh ginger, the results were not entirely as expected, with multiple ZnO morphologies present on BC support. ZnO NPs exhibit either a flower-like morphology, aggregated and somewhat disordered, while BC is visible in the background as a dense network of thin, interconnected fibres (Figure 7c), or attached bead-like entities, appearing as small white pearls closely spaced along the fibres (Figure 7d). For the BC-ZnO-GU sample, another distinct morphology occurs compared to the fresh counterpart: the fibre network remains intact, while the attached phase appears as irregular micron-sized plates closely attached to the support and are sometimes overlapping or merging (Figure 7b), indicating again a different interaction between ZnO and the dried ginger extract. Going to the bay leaf extracts, the fresh plant induces agglomerated sheet-like particles that form vertical walls on BC membrane, but also a special spatial arrangement of them in the form of an hourglass, oriented along a specific direction due to thermodynamic considerations (Figure 7e). The BC-ZnO-DU sample shows a very different aspect, with ZnO being developed as a sheet-like arrangement and creating bridges or plateaus between neighbouring fibres (Figure 7f), morphology that could be favourable for cell adhesion. For the composites prepared with rose hip extracts, new and interesting morphological details can be observed: perforated sheet-like entities, thicker compared to the previous case, embedding the BC fibres in a continuous oxide layer for the BC-ZnO-M sample (Figure 7g), and units resembling flakes made up of stacked ribbons, giving a fibrous appearance (Figure 7h). It can be concluded that the single immersion approach leads to an uncontrolled synthesis, as evidenced by the diversity of morphologies obtained, either specific to a particular experimental condition or occurring in combination within the same sample. Thus, each extract influences the amount, size, and shape of ZnO phase precipitated on BC network, as well as its arrangement and orientation, as a consequence of the different compositions of the plant extracts. Each extract contains a unique combination of phytochemicals, such as polyphenols, flavonoids, organic acids, and sugars, which can act as reducing agents, capping agents, or stabilizers during nanoparticle formation. Therefore, the chemical diversity of the extracts determines whether the NPs form beads, flakes, plates, sheets, or flower-like structures, highlighting the crucial role of biomolecules in *green* synthesis control. It is also possible that the high fibre density of BC, influencing the porosity and wettability, contributed to the uncontrolled synthesis as well, determining such variation in NP forms.

**Figure 8:**
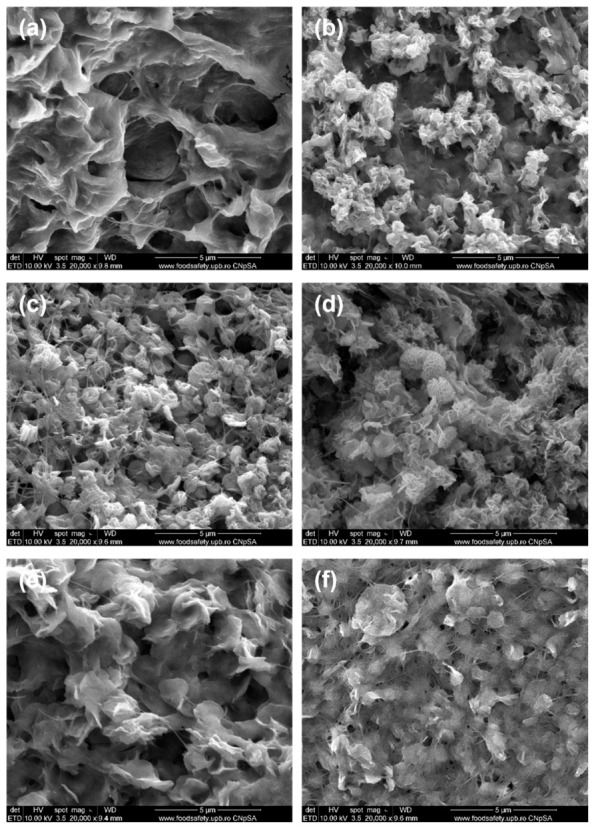
SEM images at 10,000× or 20,000× magnification of the samples in the second series: (**a**) BC-ZnO-G, (**b**) BC-ZnO-GU, (**c**) BC-ZnO-D, (**d**) BC-ZnO-DU, (**e**) BC-ZnO-M, and (**f**) BC-ZnO-MU.

Changing the synthesis procedure to a successive immersion led to different outcomes. BC was first immersed in the Zn^2+^ solution overnight and only afterward in the plant extract. The morphology of the second series of samples is quite similar to each other, and the distribution of material across the BC network is much more homogeneous compared to the first series. Figure 8a displays a denser and more compact appearance, with aggregated particles and sharper edges, forming a wavy or wrinkled ZnO layer in the BC-ZnO-G composite. In contrast, Figure 8b reveals a rougher yet finer surface for the BC-ZnO-GU sample, where sub-micron ZnO globules are loosely distributed, exhibiting a heterogeneous coverage across the BC surface. In the case of bay leaf extracts, the resulting materials contain particles agglomerated into flower-like entities on the fibres or interspersed among the fibres, with high porosity and fairly uniform distribution (Figure 8c,d). For the BC-ZnO-M and BC-ZnO-MU samples, the modified synthesis method strongly influenced the adhesion of ZnO particles to the BC support. The morphology is denser and more compact, with higher loading, and a more homogeneous and uniform distribution. The ZnO deposit sometimes appears as platelets (Figure 8e) and, at other times, as globes (Figure 8f). In any case, regardless of the adopted morphology, ZnO precipitation in the case of successive immersion is clearly favoured, most likely due to the long time of maintenance in the Zn^2+^ solution (allowing in-depth slow diffusion, even for the BC denser network), which allowed the attachment of the ions of interest to the fibre surface based on interactions with the hydroxyl (OH^−^) groups from BC.

**Figure 9:**
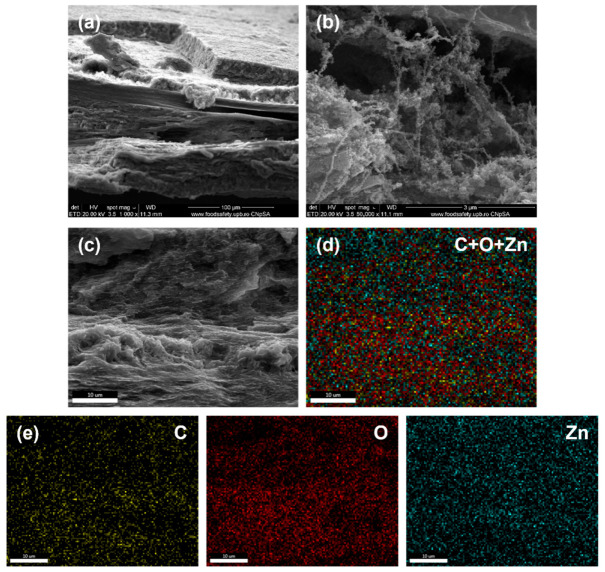
(**a**,**b**) SEM images at 1000× and 50,000× magnification of the cross-section of the BC-ZnO-GU sample in the second series, depicting the internal morphology. (**c**) Representative SEM image and (**d**) associated elemental mapping of the BC-ZnO-GU sample in the second series. (**e**) Maps for C distribution, O distribution, and Zn distribution, confirming the spatial distribution of the components within the sample.

To demonstrate the penetration of ZnO NPs into the entire volume of BC, the characterization of the obtained samples was also carried out in cross-sections. The BC-ZnO-GU composite in the second series was chosen and subjected to a cooling treatment by immersion in liquid nitrogen. This step aimed to embrittle the structure of the sample, facilitating a controlled fracture without mechanical deformations, necessary to obtain a cross-section suitable for SEM observation. The distribution of chemical elements in the fresh fracture was investigated by elemental mapping using EDX analysis. The images shown in Figure 9 evidence first the layered and porous morphology of the BC membrane, demonstrating the three-dimensional structure, and then how the ZnO NPs formed are trapped on the BC fibres in the form of laced entities, in the depths of the composite sample. This observation confirms the fact that the spatial constraints generated by the dense BC network limit the growth of ZnO NPs. The elemental mapping performed indicates a homogeneous presence of zinc (Zn) throughout the investigated area, demonstrating that the penetration of the oxide phase into the entire volume of BC support was highly efficient.

**Figure 10:**
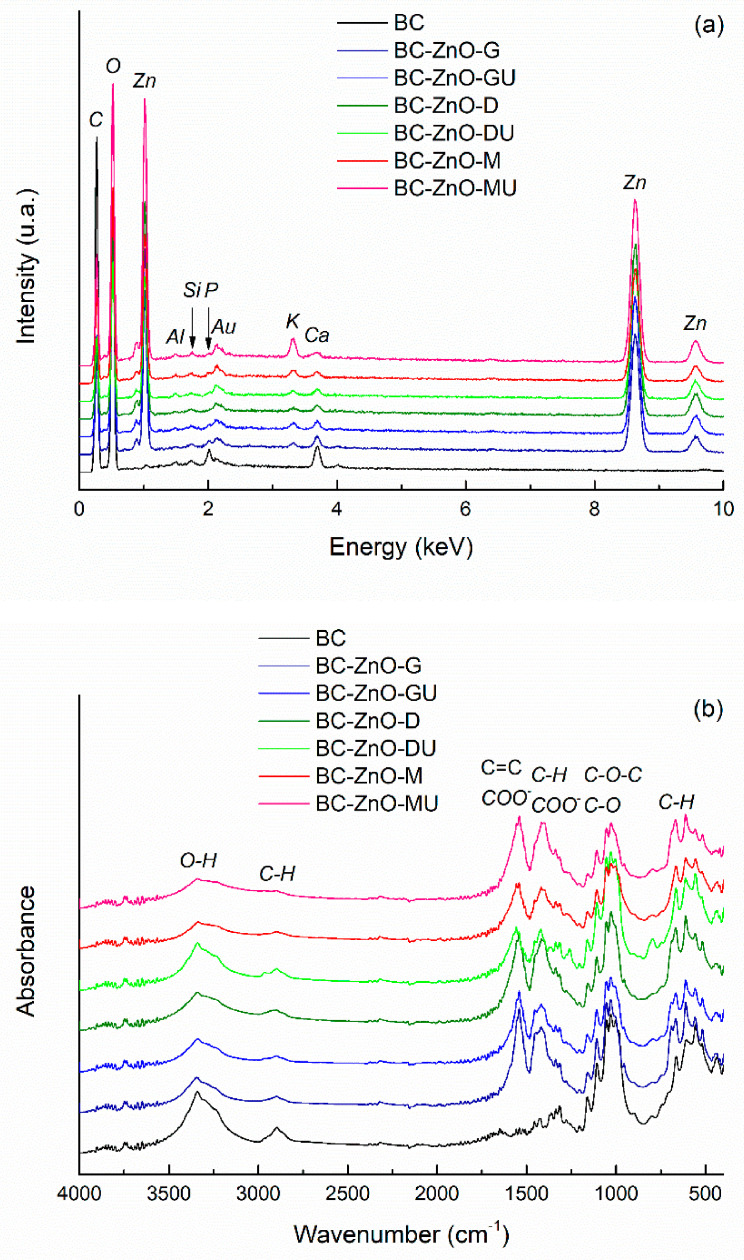
(**a**) EDX spectra and (**b**) FTIR spectra of BC-ZnO composites in the first series.

**Figure 11:**
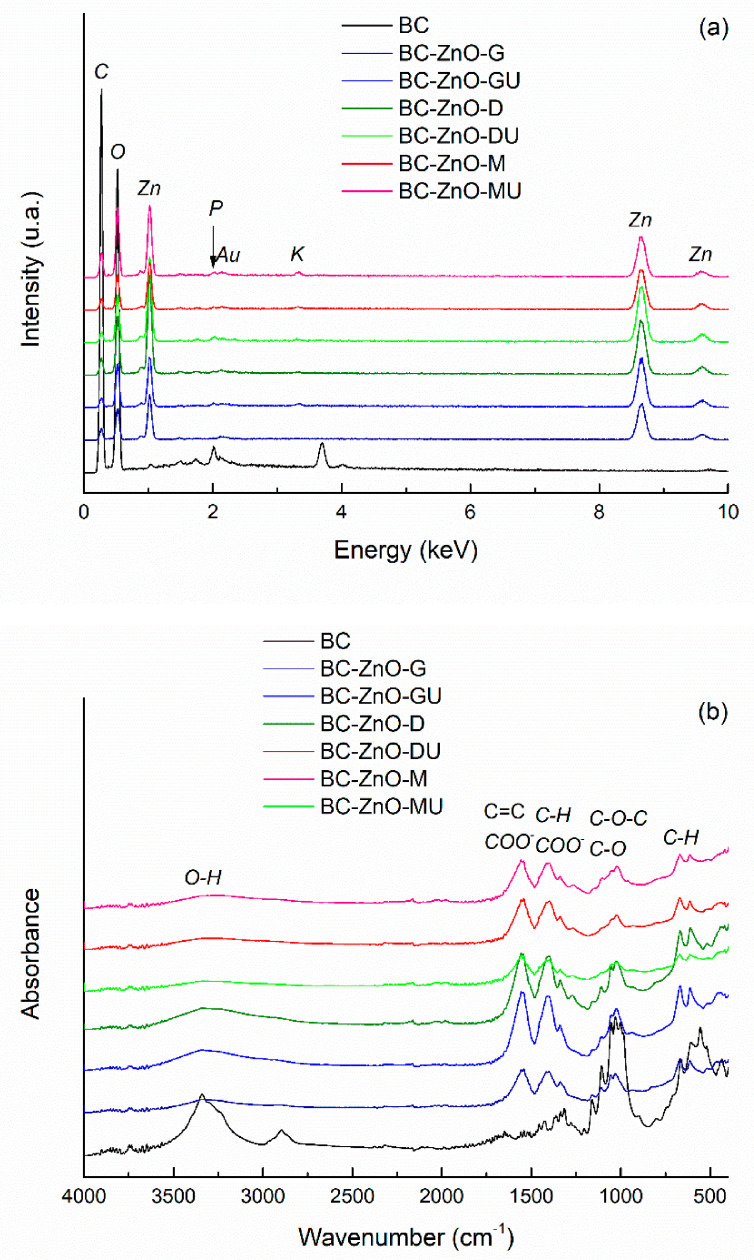

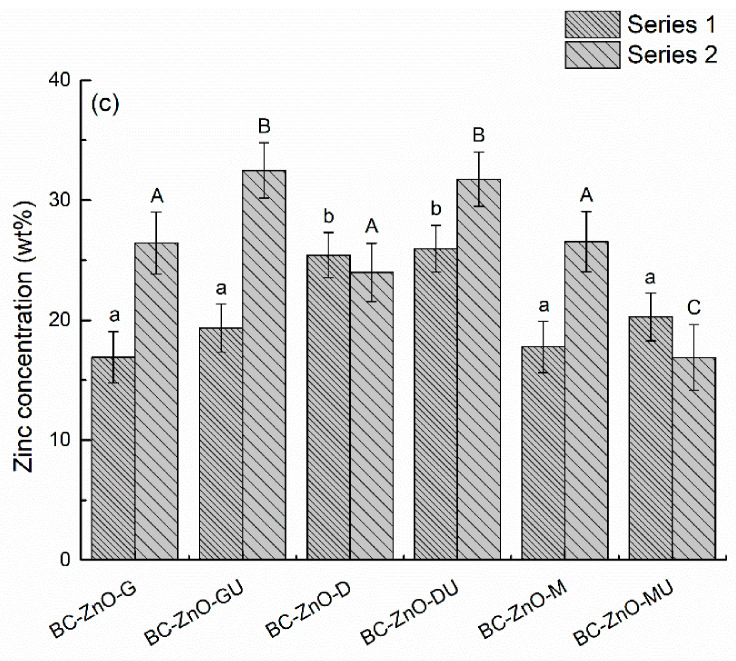
(**a**) EDX spectra and (**b**) FTIR spectra of BC-ZnO composites in the second series, revealing elemental composition and functional groups. (**c**) Comparative representation of zinc content, expressed as weight percentage, for the first and second sample series. Each bar shows the *mean diameter* and *standard deviation*. Different lowercase/uppercase letters indicate statistically significant differences between the samples (ANOVA, *p*  <  0.05).

The EDX spectrum of pure BC in Figure 10a displays characteristic lines for carbon (C) and oxygen (O), as expected. Similar results were reported for Membracel^®^, proving purity and structural integrity [56]. Moving to the EDX spectra of the composites, additional lines emerge for all experimental conditions: zinc (Zn) with the highest intensity, but also small contributions of potassium (K), calcium (Ca), silicon (Si), and phosphorus (P). The last ones originate from the BC growth medium, which usually contains ions like Na^+^, K^+^, Mg^2+^, Ca^2+^, P^5+^ in different types of phosphates, etc. These play a key role in maintaining cellular activity and supporting BC synthesis. Their presence shows that the washing step was less efficient compared to the second series, where such chemical elements are not observed in the EDX spectra (Figure 11a). To gain a deeper understanding of the quantitative implications associated with different experimental approaches (single immersion versus successive immersion), the zinc concentration was estimated from the corresponding EDX spectra, with the obtained values being presented comparatively in Figure 11c. All data fall within the 15–35 wt% range, representing an average content determined over relatively large surface areas of each composite. Notably, most samples in the second series exhibit slightly higher zinc proportions, which is consistent with the extended immersion time in the Zn^2+^ precursor solution. However, it should be emphasized that EDX spectroscopy provides only semi-quantitative results, with the final values being strongly influenced by factors such as the selected investigation area, the effectiveness of characteristic X-ray emission, as well as the relatively large uncertainties associated with low concentration levels.

In terms of the FTIR spectra, a combination of the BC fingerprint and the vibrational bands generated by the plant extracts is observed (Figure 10b and Figure 11b). Thus, the intense band at ~1025 cm^−1^ is assigned to C–O stretching vibrations of secondary alcohol groups in glucose units, whereby the one at 1050 cm^−1^ corresponds to C–O and C–C stretching vibrations within the polysaccharide backbone, and the one at 1100 cm^−1^ is related to the asymmetric C–O–C stretching vibrations of the *β*-1,4-glycosidic linkage between glucose units. Moreover, a broad and intense band emerges at ~3340 cm^−1^ for O–H stretching vibrations, being associated with hydroxyl groups and intermolecular hydrogen bonding, while, at ~2900 cm^−1^, C–H stretching vibrations of aliphatic –CH and –CH_2_ groups occur. It is obvious that the formerly mentioned bands for C=C bonds or carboxylate (COO^−^) groups in the case of pure ZnO powders (Figure 5b) appear in the case of the composites as well, which denotes that, compared to the control BC, in the composite samples there are traces remaining from the extracts used. The second series of samples maintain the same vibrational trends, with a slight decrease in the intensity of the typical BC bands, which could be explained by a shielding effect exerted by the ZnO layer preferentially deposited on the surface. At wavenumber values below 500 cm^−1^, even a shallow contribution of ZnO can be detected through the vibrations specific to the Zn–O bonds [57].

**Figure 12:**
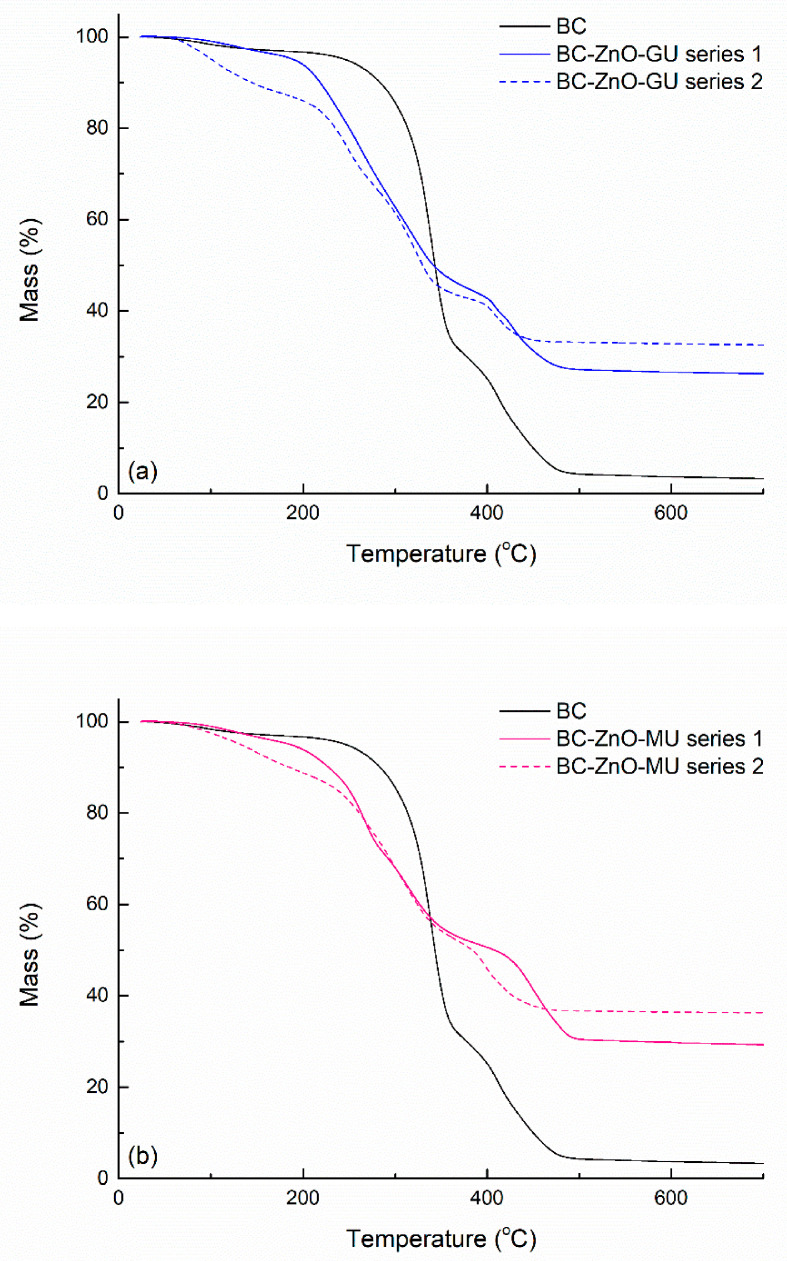
Mass variation curves for pure BC and composites in the first and second series obtained by using: (**a**) extracts of dried *Ginger rhizomes* and (**b**) extracts of dried *Rose hips*, evidencing differences in mass variation behaviour among samples.

Thermal analysis was performed for pure BC and for the composite samples in the first and second series, in which extracts from dried *Ginger rhizomes* and dried *Rose hips* were used. Figure 12 presents the recorded mass variation as a function of temperature; the plotted curves reveal a continuous decrease in mass from room temperature up to ~500 °C. For BC, the weight loss occurs in stages, starting with the elimination of water molecules trapped in the fibrillar matrix of BC, continuing with BC skeleton fragmentation and ending with the complete oxidation of the carbonaceous mass [58]. After the heat treatment, the combustion residue for the BC sample is ~2%.

The composite samples follow a similar thermal degradation path, but the weight loss stages occur at temperatures slightly shifted towards lower temperatures or with modified rates for the process evolution. The BC-ZnO-GU and BC-ZnO-MU samples in the second series show a greater mass loss at lower temperatures, indicating its ability to retain a greater amount of water in the structure. Above 200 °C, all samples show the most pronounced decrease on the curves by multi-step processes. This could be caused by the existence of several organic substances, BC, and compounds from the plant extracts, or it could be attributed to the interactions between BC and ZnO, with polymer chains interacting with ZnO and polymer chains not interacting with ZnO or possibly both. Also, the difference in temperature at which thermal degradation begins between pure BC and composite samples could result from the catalytic activity of ZnO NPs, which favour a faster disintegration of the carbon network [59]. Going from the first series to the second, the residual mass increases from ~25% to ~32% (*Ginger rhizomes*) (Figure 12a) and ~29% to ~36% (*Rose hips*) (Figure 12b), respectively, demonstrating the higher amount of ZnO embedded in the BC matrix, but also the existence of a considerable oxide phase compared to pure BC. Moreover, at the end of the combustion process (900 °C), it is found that in the case of the second series, a higher percentage of residues is present with ~6%, with these residues being represented by the remaining ZnO. This can be explained by the synthesis method used, and BC immersion time in the Zn^2+^ solution having a significant impact, thus explaining the higher loading and the higher percentage of residues for such experimental conditions [60].

**Figure 13:**
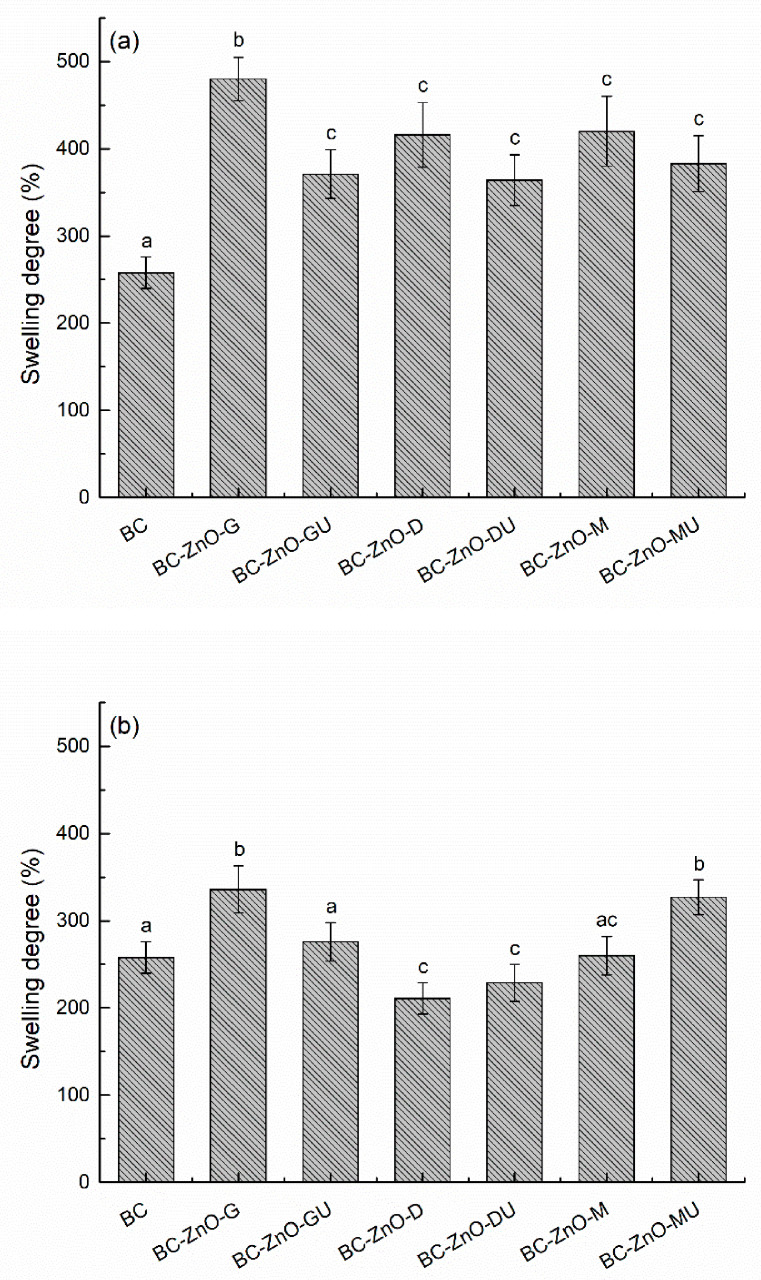
Swelling degree for pure BC and composites in the: (**a**) first and (**b**) second series, showing differences in water uptake behaviour between samples. Each bar shows the mean diameter and standard deviation. Different letters indicate significant differences between the samples (*p* < 0.05).

The evaluation of swelling capacity is essential for wound dressings, as exudate absorption is a crucial condition in supporting the healing process, especially in the case of chronic wounds. Therefore, in the present study, it was desired to preserve the high hydrophilic properties of the samples based on BC membrane, as shown in Figure 13. After the first 5 min of immersion in water, a sudden water retention was observed, and then the degree of swelling remains quite constant over time for both series. For pure BC, it has been shown that, by drying the material, the specific three-dimensional structure is affected, which leads to a significant reduction in its absorption capacity. Nevertheless, the obtained swelling degree is significantly higher than reported for Membracel^®^ (which is ~70%), indicating suitability for moderate to highly exuding wounds. The lower swelling capacity of Membracel^®^ could be related to its lower fibre density, directly influenced by cultivation conditions, large scale production, and intrusive purification [56].

In the composite samples, ZnO NPs, but also the compounds from plant extracts, led to the maintenance of a good hydration capacity of the materials designed to obtain dressings; the extracts used and oxide precipitated maintain local hydration or prevent the complete collapse of the three-dimensional network. In the case of the first series, pure BC presents the lowest degree of swelling (~258%), with the other samples having values close to each other and higher (up to ~480% for BC-ZnO-G) than the control BC (Figure 13a). In the case of the second series, the results are around the swelling degree of pure BC, due to the more controlled deposition of ZnO NPs during synthesis. The BC-ZnO-D and BC-ZnO-DU samples show a slightly lower swelling degree compared to pure BC (~211% and ~229%), while the BC-ZnO-M sample maintained its first position in the hierarchy (~336%) (Figure 13b). The differences in the swelling capacity of the samples can be attributed to the synthesis method, the amount of ZnO NPs formed, and the interaction of the compounds in the extracts with the BC network that influence this swelling property, or can be attributed to local differences in homogeneity recorded at the composite level, both on the surface and in volume [61,62].

**Figure 14:**
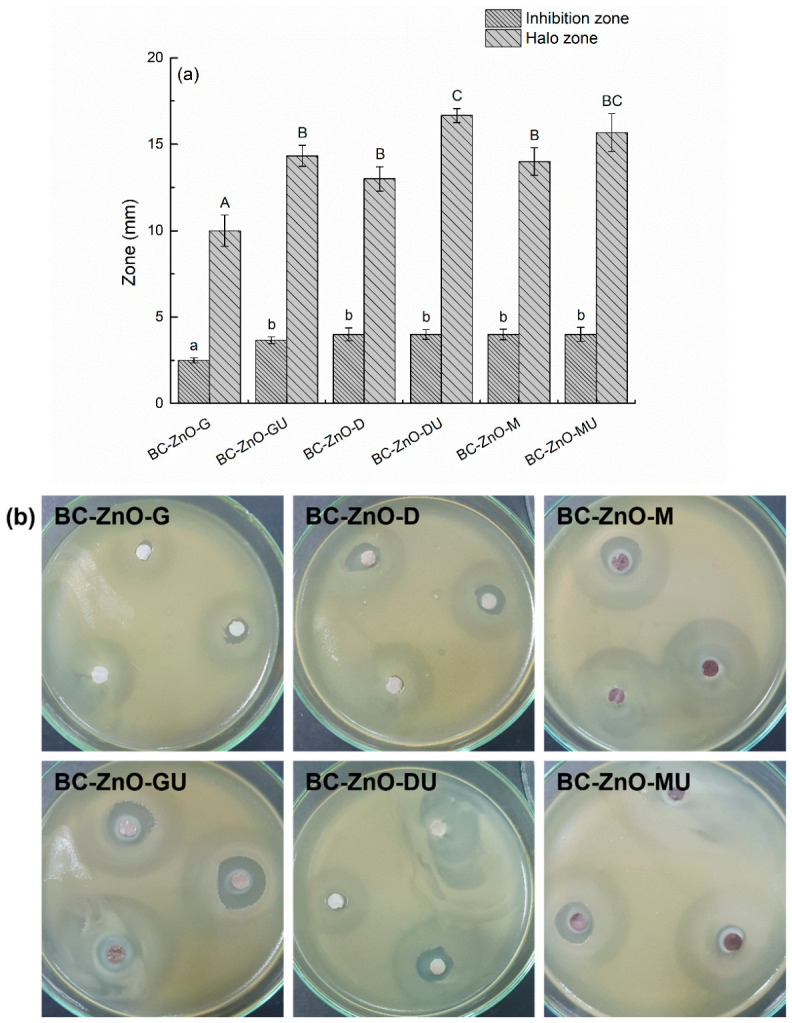
Antibacterial activity results for the composites in the first series against the *Gram*-negative bacterium *E. coli*, evaluated by the inoculum depletion method: (**a**) values of the inhibition and halo zones and (**b**) digital images showing the antibacterial performance. (**a**) Each bar shows the *mean diameter* and *standard deviation*. Different lowercase/uppercase letters indicate statistically significant differences between the samples (ANOVA, *p*  <  0.05).

The antimicrobial activity results of the inhibition and attenuation zones are presented in Figure 14. In this case, the lack of control in the synthesis of the samples demonstrates that it is difficult to obtain an ideal model, with the results being quite different from one sample to the other, thus making it difficult to conclude which of them is the optimal variant. The observed discrepancy between the small clear inhibition zone (~4 mm) and the much larger halo (~15 mm) can be attributed to differences in diffusion behaviour and antimicrobial mechanisms within the tested system. ZnO NPs, especially when immobilized on a BC matrix, exhibit limited diffusion in the working medium, resulting in a confined area of complete bacterial inhibition near the sample. In contrast, bioactive compounds from the plant extracts (*Ginger rhizomes*, *Bay leaves*, and *Rose hips*) can diffuse more readily, creating a broader area of partial bacterial growth inhibition. This leads to a concentration gradient, where strong bactericidal effects occur close to the material, while weaker, bacteriostatic effects extend further outward. Consequently, the large halo reflects the combined and diffusive action of phytochemicals, whereas the smaller clear zone indicates the localized, more potent antimicrobial activity of the ZnO phase. Digital images of bacterial biofilm development on the composite samples in the first series are also displayed in Figure 14.

**Figure 15:**
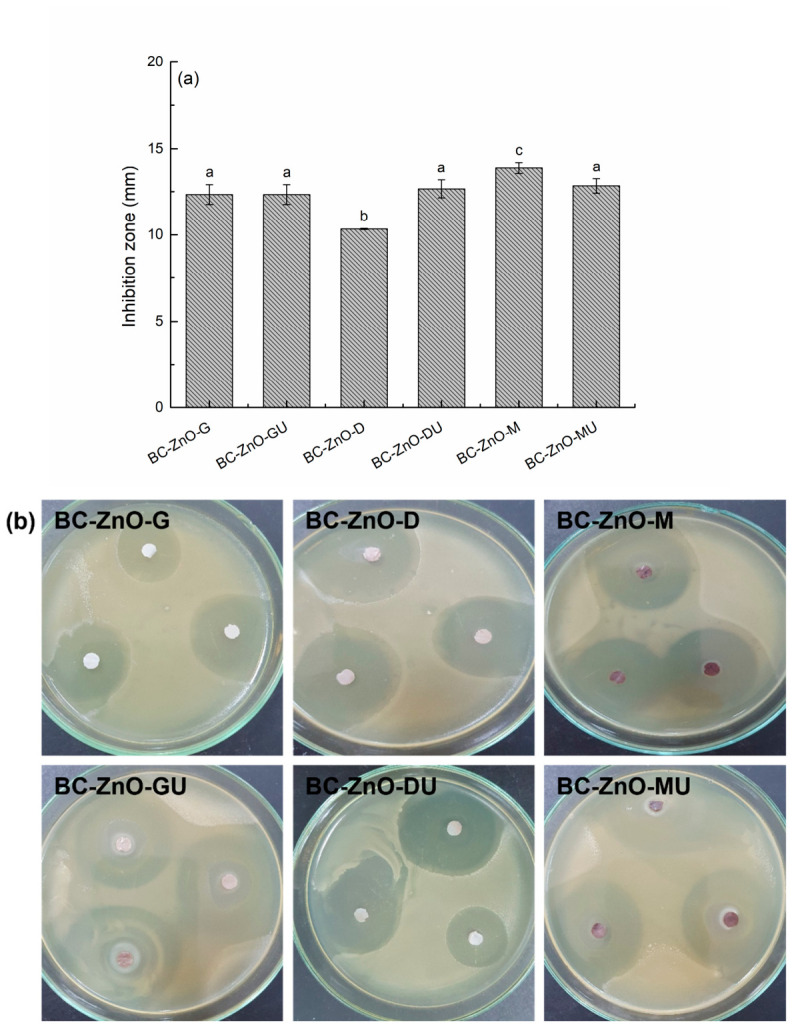
Antibacterial activity results for the composites in the second series against the *Gram*-negative bacterium *E. coli*, evaluated by the inoculum depletion method: (**a**) values of the inhibition zone and (**b**) digital images showing the antibacterial performance. (**a**) Each bar shows the *mean diameter* and *standard deviation*. Different letters indicate statistically significant differences between the samples (ANOVA, *p* < 0.05).

The result of the antimicrobial activity in the case of the samples in the second series is expressed only by the zone of inhibition and is represented by the clear zone that differentiates around the composites impregnated with the active phases, considering the real surface of the specimen (Figure 15). After comparing the values, it is observed that a good control of the synthesis process and a high efficiency of ZnO NPs deposition were achieved. Thus, through the values in the range 10–14 mm and the corresponding digital images, it is demonstrated that the samples in the second series, for which the immersion time in the Zn^2+^ solution was changed, thus obtaining a larger amount of ZnO NPs, also have a higher antibacterial activity compared to the samples in the first series.

ZnO structures within the composites exhibit antibacterial activity through a variety of mechanisms. Among these are direct interactions with the bacterial cell membrane, which can lead to its damage and, implicitly, to the destruction of the cell. One of the observed effects is the accumulation of Zn^2+^ ions at the membrane surface, generating electrostatic imbalances that can lead to deformation and affect the normal functioning of the cell. Moreover, Zn^2+^ ions slowly released from ZnO NPs penetrate bacterial cells, affecting proteins and division processes, as well as cellular respiration by interfering with the electron transport chain [63]. Also, the penetration of ZnO NPs inside the cell can negatively influence metabolic processes, leading to disturbances in energy production. In addition, ZnO generates reactive oxygen species (ROS), such as superoxide, hydrogen peroxide, and hydroxyl radicals, which attack the bacterial membrane and genetic material, compromising cellular integrity. It is important to note that a larger contact surface facilitates the generation of a larger number of ROS, thus enhancing the antibacterial effect. In this context, BC proves to be an effective support for the uniform dispersion of ZnO particles, which contributes to increasing the active surface area and enhancing antimicrobial activity.

Urge et al. [17] obtained ZnO NPs using garlic and ginger extracts, and the antibacterial properties were evaluated on some *Gram*-negative and *Gram*-positive bacteria. ZnO NPs obtained in the presence of ginger extract demonstrated higher efficacy against *Gram*-positive than *Gram*-negative bacteria. It was found that NPs synthesized from the mixture of garlic and ginger extract had a higher inhibition zone than when they were taken separately. The results suggested that NPs synthesized with mixtures of two plants may be more effective against *Gram*-negative bacteria, possibly due to the presence of a higher number of phenolic compounds and rare secondary metabolites found in both ginger and garlic.

Katepetch et al. [60] investigated the antibacterial activity of ZnO NPs incorporated into BC as well. The method used was the disk diffusion and colony counting method, against *Gram*-negative (*E. coli*) and *Gram*-positive (*S. aureus*) bacteria. The results showed that BC with ZnO NPs presents an inhibition zone for *S. aureus* of 2.9 ± 0.75 mm and *E. coli* of 5.7 ± 0.29 mm. In contrast, pure BC had no zone of inhibition. The results suggest that *E. coli* is more sensitive to ZnO NPs than *S. aureus*, due to differences in morphology of the cellular wall. The article concluded that ZnO NPs incorporated into BC demonstrates effective antibacterial activity, especially against *Gram*-negative bacteria. Moreover, all the samples in the second series of this work showed a larger inhibition zone compared to the reference article, also due to synergistic effect of the active substances with antibacterial role in the extracts used to generate ZnO NPs.

### 3.3. Composites Biocompatibility

**Figure 16:**
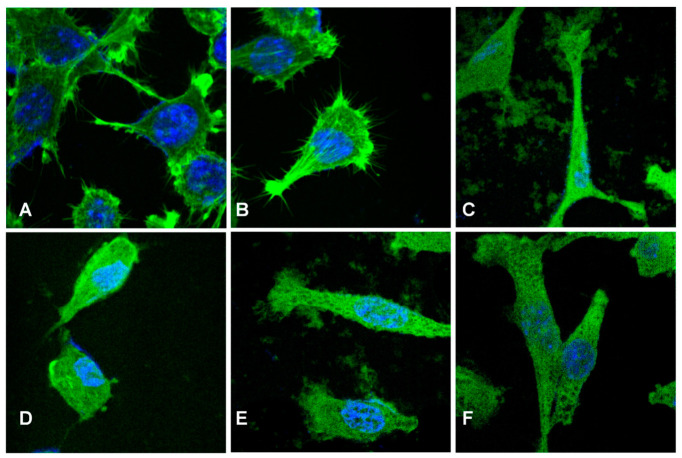
Cells’ morphological changes evidenced by fluorescence microscopy: (**A**) control, (**B**) BC, (**C**) BC-ZnO-DU series 2, (**D**) BC-ZnO-GU series 1, (**E**) BC-ZnO-GU series 2, and (**F**) BC-ZnO-MU series 2.

The samples’ biocompatibility was evaluated using fluorescence microscopy. Control cells grown on glass are shown in Figure 16A. The morphology is the normal one for the cells, namely an elongated cell morphology exhibiting clear actin filaments, long filopodia, and some intercellular connections. A normal, oval nuclear morphology is also observed, with no alterations. Similar characteristics can be seen for the cells grown in the presence of BC (Figure 16B), suggesting that the sample does not alter cell morphology and can promote cell migration through active cytoskeletal remodelling. Cells in Figure 16C were grown on the BC-ZnO-DU sample in the second series and small changes in their morphology can be identified: cells exhibit an elongated body with strong actin fibres and filopodia present. Compared to control cells, when grown on the BC-ZnO-GU sample in the first series (Figure 16D), a significant change in their morphology can be seen: cells become more rounded and a reduction in actin fibre organization is observed, with less defined protrusions, revealing a collapse of the cytoskeleton and possible damage to the cells. For the cells grown on the counterpart sample in the second series (Figure 16E), elongated cells with less organized actin filaments are present, indicating a possible alteration due to the support. Finally, in the case of the BC-ZnO-MU sample in the second series (Figure 16F), the cells keep an elongated morphology, with a well-defined actin structure. For all conditions, the cells present nuclear integrity with an elongated form and no visible damage. Overall, except for the BC-ZnO-GU sample in the first series, which induces some injury to the cells through cytoskeleton disorganization and cell rounding but with no nucleus alteration, the other samples promote cell spreading and structural integrity with good cytoskeleton organization. This distinct behaviour can be correlated with the influence of the processing route; the previously mentioned sample, which exhibited the lowest degree of biocompatibility, was the only one obtained via a single immersion in the mixture solution (Zn^2+^ solution and plant extract). This approach is relatively uncontrolled and likely promotes the retention of a high concentration of biomolecules from the extract, which may subsequently exert cytotoxic effects.

## 4. Conclusions

The current study proposes the synthesis and characterization of composite materials based on bacterial cellulose (BC) and zinc oxide (ZnO), with the latter embedded in the cellulosic matrix through a *green* approach involving plant extracts (*Ginger rhizomes*, *Bay leaves*, and *Rose hips*) obtained via the Soxhlet method. During synthesis, the bioactive compounds from the extracts acted not only as natural precipitating and capping agents, but were also retained in considerable amounts on the surface of the formed oxide nanoparticles, thereby synergistically enhancing the antimicrobial response of the resulting dressings and potentially accelerating the wound healing process. Furthermore, both single and successive immersions of pure BC in the precursor solutions (Zn^2+^ solution and plant extracts) were investigated to elucidate how processing conditions influence the final morphology and functional properties.

The characterization of the resulting samples was carried out from a physicochemical and biological point of view. The analyses confirmed the successful integration of ZnO nanoparticles into the BC matrix, as well as the presence of residual compounds from natural extracts. SEM revealed an uncontrolled and non-uniform deposition of NPs in the first case, but, for the second case, the efficiency of changing the immersion time of BC in the Zn^2+^ solution was demonstrated, obtaining a balanced deposition, with a good spatial distribution and NPs of the same shape and size. Also, SEM analysis in cross-section demonstrated the penetration of ZnO nanoparticles throughout the sample volume. EDX and XRD investigations confirmed the chemical composition and specific crystalline structure of ZnO, especially for the ginger rhizome and bay leaf-derived samples, while FTIR analysis provided information on the molecular interactions between the polymer matrix and ZnO, as well as the phytochemicals from the plant extracts. Thermal analysis provided valuable information, demonstrating that, indeed, the samples in the second series embed a greater amount of ZnO NPs, the combustion residue being represented by the thermally stable inorganic phase.

Of particular importance, the antibacterial evaluation was decisive, being performed against the *Gram*-negative bacteria *Escherichia coli* and showing a significantly increased activity for the samples in the second series compared to those in the first series, due to the higher amount of ZnO NPs deposited in the three-dimensional network of BC. These results support and confirm the antibacterial property of ZnO and the fact that, together with natural extracts, they can act synergistically to enhance the antimicrobial effect of the obtained dressings. The largest inhibition zone was found for the BC-ZnO-M sample in the second series, thus demonstrating that the ideal variant for obtaining dressings is the one with fresh rose hip extract. This could also be related to the crystalline characteristics of ZnO obtained in this way, the XRD pattern having a modified shape because of an altered structure in terms of order, orientation, or stoichiometry. The differences between plant extracts obtained from fresh and dried material of the same plant were not significant, as both displayed comparable performances, indicating that the extraction process is equally effective regardless of the initial plant state.

In conclusion, the present work successfully demonstrates, at the laboratory level, the possibility of obtaining effective antibacterial dressings through *green* synthesis, using plant extracts, ZnO, and BC as a support, highlighting the special properties of the materials used, thus being an ideal alternative to current treatments and an innovation for regenerative medicine.

To improve the synthesis method and explore extended functional properties of the dressings, a future direction could involve the use of unconventional technologies such as microwave or ultrasound irradiation to accelerate the synthesis process and for a more uniform distribution of ZnO nanoparticles in the BC network. These methods could reduce the reaction time and improve the morphological characteristics of the final composites. Also, the addition of a third functional phase, such as silver nanoparticles or drugs (local antibiotics or substances to accelerate healing), could be investigated, with the aim of obtaining multifunctional dressings with antibacterial, antioxidant, and controlled release properties of active substances. The integration of these new directions could lead to the development of advanced dressings, tailored for specific clinical applications.

## Figures and Tables

**Table 1:** Moisture content of vegetal materials under fresh and dried conditions prior to extraction, together with the solid–liquid ratios applied in the extraction experiments and the associated extract codes.

Vegetal Material	Condition	Moisture Content (%)	Solid–Liquid Ratio	Extract Code
*Ginger rhizomes*	Fresh	88.9 ± 0.9	1:15	G
	Dry	8.5 ± 0.1	1:30	GU
*Bay leaves*	Fresh	51.7 ± 1.4	1:30	D
	Dry	7.5 ± 0.3	1:75	DU
*Rose hips pericarp*	Fresh	64.1 ± 1.2	1:15	M
	Dry	5.2 ± 0.1	1:15	MU

## Data Availability

The raw data supporting the conclusions of this article will be made available by the authors on request.
